# 

**Published:** 1873-12

**Authors:** 


BOOKS RECEIVED—Which will be reviewed in future numbers of the JOURNAL :

THE PRACTICE OF SURGERY. By Thomas Bryant, F.R.C.S., Surgeon to Guy's Hospital. Henry C. Lea : Philadelphia. 1873.

ON THE MECHANICAL TREATMENT OF THE DISEASES OF THE HIP-JOINT. By Charles Fayette Taylor, M.D. William Wood & Co. : New York. 1873.

LACERATION OF THE PERINEUM AND VESICO VAGINAL FISTULA. By D. Hayes Agnew, M.D. Lindsay & Blakeston : Philadelphia. 1873.

LECTURES ON DISEASES AND INJURIES OF EAR. Delivered at St. George's Hospital. By W. B. Dalby, F.R.C.S., M.B., Cantab. Lindsay & Blakeston : Philadelphia. 1873.

INTRODUCTORY TO THE STUDY OF CLINICAL MEDICINE. By Octavius Sturges, M.D., Cantab. Henry C. Lea : Philadelphia. 1873.

HAND-BOOK OF PHYSIOLOGY. By William Stenhouse Kirkes, M.D. Henry C. Lea : Philadelphia. 1873.

TRANSACTIONS OF THE AMERICAN MEDICAL ASSOCIATION. Vol. 24. 1873.

AN INTRODUCTION TO PRACTICAL CHEMISTRY, INCLUDING ANALYSIS. By John E. Bowman, F.C.S. Henry C. Lea : Philadelphia. 1873.

A TREATISE ON THE DISEASES OF THE EYE. By J. Soelberg Wells, F.R.C.S. Henry C. Lea : Philadelphia. 1873.

A MANUAL OF MEDICAL JURISPRUDENCE. By Alfred Swaine Taylor, M.D., F.R.S. Henry C. Lea: Philadelphia. 1873.

CHEMISTRY, INORGANIC AND ORGANIC, WITH EXPERIMENTS. By Charles Loudon Bloxam, Prof. of Chemistry, in King's College, London. Henry C. Lea: Philadelphia. 1873.

A HAND-BOOK OF THE THEORY AND PRACTICE OF MEDICINE. By Frederick T. Roberts, M.D., B.S.C., M.R.C.P. Lindsay & Blakeston: Philadelphia. 1873.

---

ACKNOWLEDGEMENTS.—Atlanta Herald, Daily; American Practitioner; Druggists' Circular; Northwestern Medical and Surgical Journal; Wood's Household Magazine; Medical News and Library; The Druggist; Detroit Review of Medicine and Pharmacy; American Newspaper Reporter; Chicago Medical Journal; Medical Record; Medical and Surgical Reporter; Medical Examiner; Methodist Advocate; American Journal Pharmacy: Boston Medical and Surgical Journal; Richmond and Louisville Medical Journal; Cincinnati Lancet and Observer; The Clinic; Il Tagliamento—Pordenone, Nov. 22, 1873; Proceedings Medical Association State of Arkansas; Discourse Commemorative, Hugh L. Hodge, M.D., L.L.D. ; Homocultology — an Essay, by "The Author."

